# Metaplastic carcinoma of the breast mimicking breast implant-associated squamous cell carcinoma: a challenging differential diagnosis

**DOI:** 10.1080/23320885.2025.2486239

**Published:** 2025-04-12

**Authors:** E. Rogges, M. M. Petrino, G. Firmani, M. Sorotos, F. Santanelli di Pompeo, A. Di Napoli

**Affiliations:** a Pathology Unit, Department of Clinical and Molecular Medicine, Sant’Andrea University Hospital, Sapienza University of Rome, Italy; b PhD School in Translational Medicine and Oncology, Department of Medical and Surgical Sciences and Translational Medicine, Faculty of Medicine and Psychology, Sapienza University of Rome, Rome, Italy; c Plastic and Reconstructive Surgery Unit, Faculty of Medicine and Psychology, Sapienza University of Rome, Department of Neuroscience, Mental Health, and Sense Organs (NESMOS), Sant’ Andrea Hospital, Rome, Italy

**Keywords:** Metaplastic carcinoma, breast Implant-associated squamous cell carcinoma, breast cancer recurrence, BIA-SCC

## Abstract

Metaplastic carcinoma of the breast (MBC) is an uncommon disease that accounts for 0.2–1% of all invasive breast carcinomas, comprising a heterogeneous group of diseases characterized by differentiation of the neoplastic epithelium to squamous cells and/or mesenchymal-looking elements. Breast implant–associated squamous cell carcinoma (BIA-SCC) is a rare complication of breast implantation, with 22 cases reported in the literature. Due to the histological overlap between MBC and BIA-SCC, the differential diagnosis may be challenging, especially in patients with an advanced cancer-bearing breast implant, in which assessing the tumor’s primary site may be difficult. The limited amount of scientific data on BIA-SCC implies the absence of a standardized diagnostic method and of a specific staging system to guide patients’ clinical management. Of the 22 BIA-SCC cases reported in the literature, 14 (64%) had squamous metaplasia of the inner surface of the capsule, whereas in 10 (45%), there was a histologically proven spread to the extracapsular tissues without a detailed description of the capsule infiltration. Herein, we describe the case of a peri-implant tumor mass with squamous histology in a patient treated with mastectomy and implant-based breast reconstruction for a microinvasive breast carcinoma, in which the absence of squamous metaplasia of the capsule and of its neoplastic infiltration favored a diagnosis of MBC likely originating from the peri-implant tissue. This case suggests that in patients with peri-implant cancers with squamous differentiation, the extension of the tumor throughout the capsule thickness and the presence of squamous metaplasia of the capsule are critical factors that should be considered in the differential diagnosis between BIA-SCC and MBC. In addition, even in cases with capsule-confined tumors, the depth of the capsular involvement can be used to stage the disease, similar to what is currently recommended for BIA-ALCL.

## Introduction

Metaplastic carcinoma of the breast (MBC) is an uncommon disease that accounts for 0.2–1% of all invasive breast carcinomas. It comprises a heterogeneous group of diseases characterized by differentiation of the neoplastic epithelium to squamous cells and/or mesenchymal-looking elements [1]. Histologically MBC is classified as epithelial-only carcinomas, pure sarcomatoid carcinomas, and biphasic epithelial and sarcomatoid carcinomas [1]. Despite this heterogeneity, the cell of origin of MBC is assumed to be a de-differentiated epithelial tumor cell or alternatively a basal-like stem cell. Both these hypotheses rely on the genetic evidence of a monoclonal origin from a common precursor cell of each tumoral component [2–5]. However, it remains unclear whether somatic mutations could cause the divergent differentiation that allows metaplastic carcinoma subtypes [6].

Breast implant–associated squamous cell carcinoma (BIA-SCC) is a very rare complication of breast implantation, with 19 cases recognized by the FDA until 2023 [7] and 22 cases reported in the literature [8–26]. Histologically, it is characterized by the formation of sheets and nests of epithelial cells with squamous differentiation. Despite the histological overlap with MBC, BIA-SCC differentiates from MBC because it derives from the malignant transformation of the squamous metaplasia occurring on the inner surface of the periprosthetic breast capsule [8,9, 21,27]. However, the differential diagnosis between these two entities can be challenging, especially when the tumor is mass-forming and occurs in patients with a history of breast cancer. We report the first case of MBC developed in a patient treated with nipple sparing mastectomy and implant-based breast reconstruction for a microinvasive breast carcinoma of no special type with an extensive high grade *in situ* ductal carcinoma component.

## Patients/materials and methods

Formalin-fixed paraffin-embedded (FFPE) tissue sections of the tumor mass were reviewed by expert pathologists by utilizing morphological and immunohistochemical criteria according to the 5^th^ edition of the World Health Organization (WHO) classification of breast tumors [1]. FFPE sections were immunostained for CK5/6, GATA3 (Biocare Medical, Pacheco, CA, USA), CK7, p40, ER, PR, HER2, CKAE1/AE3 (Leica Biosystems, Wetzlar, Germany) p63, and Ki67 (Dako, Agilent, Santa Clara, CA, USA) using an automated immunostainer (Bond-III, Leica Biosystems, Wetzlar, Germany). *In situ* hybridization for Epstein-Barr virus (EBV) -encoded mRNA (EBER) was performed on paraffin sections using BOND EBER Probe (Bond-III, Leica Biosystems, Wetzlar, Germany).

## Results

**Figure 1.**
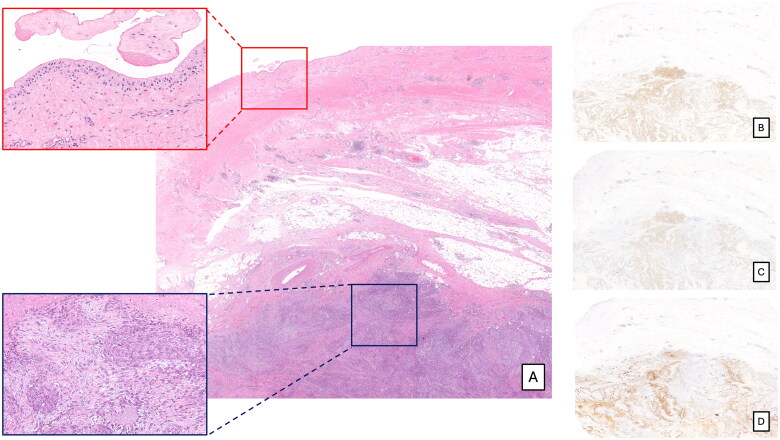
Histological examination revealed a mass-forming lesion distant from the luminal surface of the peri implant capsule and infiltrating the adipose tissue but not the periprosthetic capsule [A, hematoxylin and eosin (H&E), optical magnification (o.m. ×6)]. The capsulewas characterized by the presence of pseudo-synovial metaplasia (A, H&E upper insert, o.m. ×100), whereas the mass was composed by solid nests of large epithelial cells, with focal spindle morphology, (A, H&E lower insert, o.m. ×200). The neoplastic cells were positive for p63 (B, o.m. ×6), p40 (C, o.m. ×6), and focally for CK5/6 (D, o.m. ×6).

A 53-year-old female was diagnosed on December 2020 with microinvasive ductal carcinoma of no special type associated with extensive high-grade *in situ* ductal carcinoma on a 5 cm mass of the central and external quadrants of the right breast. The patient underwent nipple sparing mastectomy with sentinel node biopsy and implant-based breast reconstruction with a textured device. Histological examination of sentinel lymph node tissue had not revealed micro or macrometastasis. TNM staging was pT1mi pN0(sn) according to the current staging system (American Joint Committee on Cancer [AJCC], 2017) [28]. The prognostic/predictive factors assessment on the tumor tissue showed positivity for estrogen (ER) and progesterone receptor (PgR) (both in 40% of the neoplastic cells), a proliferation index (Ki67) of 25% and human epidermal growth factor receptor 2 (HER2) score 1+ (negative). Hormone therapy was started after surgery. Three years later, the patient presented with a right chest wall solid mass. Fluorodeoxyglucose (FDG) computed tomography (CT) and positron emission tomography (PET) scans revealed a 10 × 7 cm mass in the soft tissue of the right lateral chest wall, in proximity (0.5 cm) to the breast implant, with a maximum standardized uptake value (SUVmax) of 11, and no other area of metabolic activity. With the suspicion of cancer relapse, she subsequently underwent surgical tumor excision with an en-bloc capsulectomy. Macroscopic examination showed a mass-forming lesion with 0.5 cm distance from the luminal surface of the peri implant capsule, corresponding histologically to solid nests of large epithelial cells, with focal spindle morphology and necrotic areas, infiltrating the adipose tissue but not the periprosthetic capsule (Figure 1). Immunohistochemically the neoplastic cells expressed p63, p40 and GATA3, CK5/6, CKAE1/AE3 and focally CK7, and were negative for ER, PgR, HER2, and EBER- (Figure 1). The proliferation index (Ki67) was 40-50%. The periprosthetic breast capsule was represented by fibrous tissue with mild chronic inflammation and diffuse pseudo-synovial metaplasia of the luminal surface without evidence of squamous metaplasia. These findings did not support the diagnosis of BIA-SCC, favoring a pure squamous MBC. The patient received adjuvant carboplatin–doxorubicin chemotherapy followed by radiotherapy. Post-chemotherapy CT scan revealed multiple lung nodules suggestive of metastatic disease, for which the patient is currently under treatment with Trop2-targeted therapy (Sacituzumab Govitecan).

## Discussion

MBC is a heterogeneous group of invasive breast carcinomas (IBCs) characterized by differentiation of the neoplastic epithelium to squamous cells and/or mesenchymal-looking elements [1]. BIA-SCC is considered a separate entity distinct from MBC with pure squamous differentiation, arising from the inner lining of the breast implant capsule. It occurs after a mean time of 22.74 years (range 11–40 years) in patients with both smooth and textured implants [8–26,29,40].

A recent paper by our group highlighted the differences between pure squamous MBC and BIA-SCC by systematically reviewing the literature data [29]. The review highlighted that the mean age of the patients diagnosed with pure squamous MBC was about seven years older (61.3 years; range, 18–99 years) than the mean age of the patients with BIA-SCC diagnosis (54.1 years; range 40–81 years). The main dissimilarity that emerges is that only 0.13% of pure squamous MBC patients received a breast implant for cosmetic augmentation, whereas in patients diagnosed with BIA-SCC, the reason for implantation was augmentation in 73.3% and reconstruction in 23.3% of the cases. Both pure squamous MBC and BIA-SCC have an aggressive behavior with locoregional lymph node metastases respectively in 25.6% and 23.3% of the patients and distant metastases in 7.6% and 30.0% involving mainly lungs and liver in both cohorts, cutaneous/subcutaneous tissues predominantly in BIA-SCC, and bones in MBC patients only [29].

MBC patients are reported to have lower response to chemotherapy and a worse clinical outcome after chemotherapy than other forms of triple-negative breast carcinomas [30–33]. The presence of high-grade spindle cell, squamous cell, high-grade adenosquamous MBC components and the presence of high number of morphologies within mixed metaplastic carcinomas correlates with a worse outcome [34–36]. The 3-year, 5-year, and 10-year overall survival rates for MBC are 77% [37], 62% [33,38], and 53% [39], respectively.

The Food and Drug Administration (FDA) reported that BIA-SCC was located in the capsule around the breast implant, often in the posterior aspect (behind the implant), without being present in the breast tissue. They also highlighted three reports of death due to the disease [7]. According to the American Society of Plastic Surgeons (ASPS), BIA-SCC can exhibit highly invasive properties, with 80% of extracapsular spread at presentation and metastasis in lymph nodes and distant sites, with a 6-month mortality rate of 43.8% [40]. Based on the small amount of data available, neoadjuvant and postoperative chemotherapy has been administrated with limited-to-no success, whereas radiation therapy has been used on a limited basis and mostly for palliative purposes [41]. The depth of capsular and extracapsular tumor invasion could be an important prognostic factor. We reviewed the 22 BIA-SCC cases reported in the literature to date [8–26], and in only 10 cases (45%), the involvement of the capsular and extracapsular tissues was histologically detailed (Table 1). This limits the possibility to develop a specific tumor staging system that can be used to guide patients’ clinical management and prognosis assessment.

**Table 1.** Clinicopathological features of BIA-SCC cases reported in the literature.

Cases	Squamous metaplasia	Extracapsular invasion	Locoregional Lymph nodes at diagnosis	Metastasis or local recurrence	Therapy	Follow-up
Paletta et al.	yes	No (histologically proven)	Negative (histologically proven)	Negative (CT)	Capsulectomy, mastectomy	Disease free 12 months after mastectomy
Kitchen et al.	yes	No (histologically proven)	NA	NA	Capsulectomy	NA
Satgunaseeian et al.	NA	No (histologically proven)	NA	NA	Mastectomy	NA
Zomerlei et al.	yes	Yes (PET)	Negative (histologically proven)	NA	Capsulectomy, mastectomy, axillary lymph node disection, chest wall resection	NA
Olsen et al.	yes	Yes (histologically proven)	Negative (histologically proven)	Subcutaneous soft tissues of the axilla, left upper arm and chest wall (histologically proven)	Mastectomy, CHT+RT, excision of axillary metastases followed by CHT+RT	Under palliative radiation therapy
Olsen et al.	yes	Yes (histologically proven)	Negative (histologically proven)	Lung and soft tissues (PET) Liver (histologically proven)	Mastectomy, CHT+RT	Died of disease
Buchanan et al.	NA	NA	Positive (PET)	Negative (PET)	Capsulectomy, mastectomy, chest wall resection and RT	Disease free 8-year after diagnosis
Resetkova et al.	NA	Yes (MRI)	NA	NA	Mastectomy and chest wall resection	NA
Zhou et al.	yes	Yes (histologically proven)	Negative (CT)	Lung nodules, kidney and liver (histologically proven), Leptomeningeal spread (NA)	Capsulectomy, re-excision of the chest wall mass and RT	Died of disease 1 year after diagnosis
Alfaro et al.	yes	NA	NA	NA	NA	NA
Goel et al.	NA	Yes (histologically proven)	Positive (CT, MRI)	NA	Mastectomy, axillary lymph node dissection, chest wall escision and CHT+RT	NA
Zikiryakhodzhaev et al.	NA	Yes (histologically proven)	Positive (histologically proven)	Negative	Chest wall resection	Disease free 9-months after diagnosis
Goldberg et al.	yes	Yes (MRI and CT)	NA	Axillary region (histologically proven) Pleural effusions not histologically defined	Partial capsulectomy and CHT	Died of disease 3 months after the diagnosis
Goldberg et al.	NA	Yes (histologically proven)	Negative (CT, PET)	Negative (CT, PET)	Capsulectomy and CHT+RT	Under palliative care, lost follow-up
Liu et al.	NA	Yes (histologically proven)	Positive (PET)	NA	Chest wall resection, prothesis removal, lymph node biopsy, CHT+RT	Disease free at 24-month follow-up
Soni et al.	yes	Yes (histologically proven)	Negative (histologically proven)	NA	Capsulectomy, mastectomy, lymph node biopsy, CHT+RT	Remission 12 months after adyuvant therapy
Whaley et al.	yes	No (histologically proven)	NA	NA	Capsulectomy	Disease free at 9-months-follow-up
Whaley et al.	yes	Yes (histologically proven)	Negative (histologically proven)	NA	Capsulectomy	Lost at follow-up
Vorstenbosh et al.	NA	Yes (CT)	NA	NA	Mastectomy, CHT and chest wall resection	Disease free over 2 years
Xia et al.	yes	Yes (PET-CT and MRI)	Negative (histologically proven)	Multiple metastasis including axillary lymph nodes, skin of the chest and pleura (NA)	Capsulectomy, sentinel lymph node biopsy and CHT+RT	Multiple metastasis one year after the diagnosis
Rosenberg et al.	yes	Yes (histologically proven)	Negative (histologically proven)	Negative (PET)	Mastectomy, capsulectomy, axillary lymph node dissection, CHT+RT	Recurrent diasease two months after the surgery
Badri et al.	yes	No (histologically proven)	Negative (histologically proven)	Negative (PET)	Mastectomy, capsulectomy, lymph node biopsy and CHT	Disease free at 6 months-follow-up

**Figure 2.**
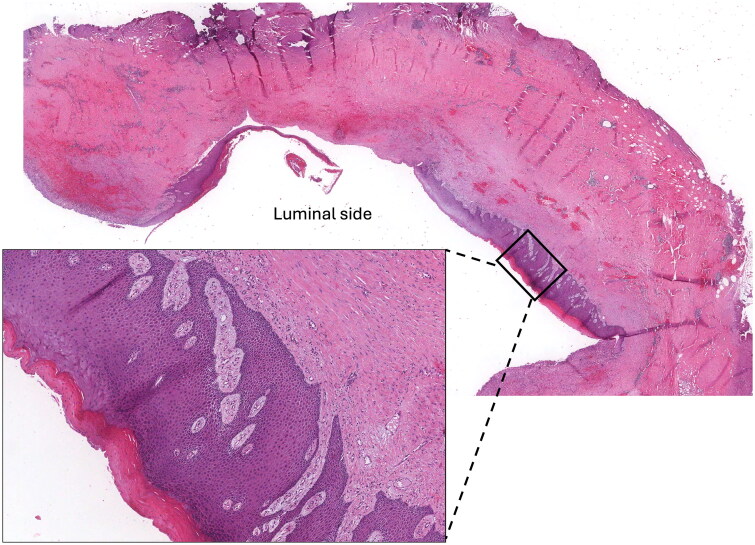
Squamous metaplasia in capsulectomy specimen removed for non-tumoral reasons (H&E o.m. ×10, lower insert ×200).

In patients with advanced disease, where the primary site of the tumor can’t be clearly identified, the differential diagnosis between BIA-SCC and pure squamous MBC may be particularly challenging. This occurs because of the close histological resemblance and the absence of disease-specific immunohistochemical markers. Both the diseases usually lack expression of ER, PgR, and HER2 [37,38, 42,43], and GATA3 may be expressed in both MBC and squamous cell carcinomas [44,45]. In our case the presence of a mass located in the fibroadipose tissue, without the involvement of the implant capsule, and the absence of areas of squamous metaplasia of the inner surface of the capsule argue against the hypothesis of a tumor originating from the capsule. Among the 22 BIA-SCC cases reported in the literature, 14 described capsular squamous metaplasia (64%) (Table 1). Squamous metaplasia is considered as a benign form of adaptation to chronic tissue injury [46], and it can be an incidental finding in capsulectomy specimens removed for non-tumoral reasons (Figure 2) [47]. While initially reversible, it may evolve as the chronic exposure to the stimulus prolongs, ultimately playing a role in the tumorigenic process, potentially making squamous metaplasia a precursor of BIA-SCC [22, 41].

The history of breast cancer in our patient may raise concerns of a possible relapse as MBC. Isolated chest wall recurrences in patients with breast cancer treated with radical mastectomy are not frequent [48]. In a 20-year follow-up of a randomized study comparing breast-conserving surgery with radical mastectomy for early breast cancer, Veronesi et al. described a similar survival rate in both groups, even though the probability of a recurrent tumor was significantly higher in the patients treated with breast-conserving therapy than in the radical-mastectomy ones (30 of 352 vs. 8 of 349 patients) [49].

There are only anecdotal cases reporting breast cancer recurrences as MBC in patients treated with mastectomy and axillar lymph node dissection [50–52]. Genetic studies proving a clonal relationship between the primary breast cancer and its MBC relapse are lacking. In our case, it seems likely that the MBC represented a possible relapse of the previous breast cancer or alternatively an unrelated tumor originating from a residual breast parenchyma.

## Conclusions

Herein, we present the first case in the literature of an MBC in a patient treated with mastectomy and implant-based breast reconstruction for a microinvasive breast cancer, in which BIA-SCC was excluded by the absence of tumor extension throughout the capsule thickness and of squamous metaplasia of the capsule. Moreover, similarly to what is currently recommended for breast implant-associated anaplastic large cell lymphoma (BIA-ALCL), we suggest to detail the depth of tumor invasion, even in capsule-confined squamous tumors, to allow an accurate staging of the disease.

## Data Availability

The data that support the findings of this study are available within the article.
